# HIF-1α/GPER signaling mediates the expression of VEGF induced by hypoxia in breast cancer associated fibroblasts (CAFs)

**DOI:** 10.1186/bcr3458

**Published:** 2013-08-15

**Authors:** Ernestina Marianna De Francesco, Rosamaria Lappano, Maria Francesca Santolla, Stefania Marsico, Arnaldo Caruso, Marcello Maggiolini

**Affiliations:** 1 Department of Pharmacy, Health and Nutritional Sciences, University of Calabria, 87036 Rende (Cosenza), Italy; 2 Section of Microbiology, University of Brescia, Piazzale Spedali Civili 1, 25123 Brescia, Italy

## Abstract

**Introduction:**

Carcinoma-associated fibroblasts (CAFs) play a pivotal role in cancer progression by contributing to invasion, metastasis and angiogenesis. Solid tumors possess a unique microenvironment characterized by local hypoxia, which induces gene expression changes and biological features leading to poor outcomes. Hypoxia Inducible Factor 1 (HIF-1) is the main transcription factor that mediates the cell response to hypoxia through different mechanisms that include the regulation of genes strongly associated with cancer aggressiveness. Among the HIF-1 target genes, the G-protein estrogen receptor (GPER) exerts a stimulatory role in diverse types of cancer cells and in CAFs.

**Methods:**

We evaluated the regulation and function of the key angiogenic mediator vascular endothelial growth factor (VEGF) in CAFs exposed to hypoxia. Gene expression studies, Western blotting analysis and immunofluorescence experiments were performed in CAFs and breast cancer cells in the presence of cobalt chloride (CoCl_2) _or cultured under low oxygen tension (2% O_2_), in order to analyze the involvement of the HIF-1α/GPER signaling in the biological responses to hypoxia. We also explored the role of the HIF-1α/GPER transduction pathway in functional assays like tube formation in human umbilical vein endothelial cells (HUVECs) and cell migration in CAFs.

**Results:**

We first determined that hypoxia induces the expression of HIF-1α and GPER in CAFs, then we ascertained that the HIF-1α/GPER signaling is involved in the regulation of VEGF expression in breast cancer cells and in CAFs exposed to hypoxia. We also assessed by ChIP assay that HIF-1α and GPER are both recruited to the *VEGF *promoter sequence and required for *VEGF *promoter stimulation upon hypoxic condition. As a biological counterpart of these findings, conditioned medium from hypoxic CAFs promoted tube formation in HUVECs in a HIF-1α/GPER dependent manner. The functional cooperation between HIF-1α and GPER in CAFs was also evidenced in the hypoxia-induced cell migration, which involved a further target of the HIF-1α/GPER signaling like connective tissue growth factor (CTGF).

**Conclusions:**

The present results provide novel insight into the role elicited by the HIF-1α/GPER transduction pathway in CAFs towards the hypoxia-dependent tumor angiogenesis. Our findings further extend the molecular mechanisms through which the tumor microenvironment may contribute to cancer progression.

## Introduction

The cooperative interactions among tumor cells and reactive stroma strongly contribute to cancer development and progression [1,2]. Cancer-associated fibroblasts (CAFs) have been indicated as the main cellular component of the tumor microenvironment involved in cancer initiation, invasion and metastasis [3-5]. In breast malignancies, CAFs exert a pivotal role in tumor progression and resistance to therapeutics through multiple mechanisms, including the stimulation of new blood vessels [6], mainly generated by a hypoxic tumor microenvironment [7-9]. Indeed, mechanisms of cell sensing and adaptation to stressful environments are activated within the hypoxic tumor mass, leading to the growth and aggressiveness of malignant cells [10]. The transcription factor Hypoxia Inducible Factor 1 (HIF-1) primarily mediates the cell responses to a low oxygen tension playing a crucial role in cancer development [11,12]. In particular, HIF-1 activates a signaling transduction network which drives the adaptation of tumor cells to hypoxic conditions towards a more aggressive cancer phenotype [13]. HIF-1 is a heterodimeric protein composed of the hypoxia-inducible α subunit and the constitutively expressed β subunit [14]. HIF-1α and HIF-1β dimerize upon exposure to hypoxia, generating a complex which binds to the hypoxia-responsive elements (HREs) located within the promoter region of target genes [15]. In this regard, it has been shown that HIF-1 is a leading regulator of tumor angiogenesis following hypoxia, as it regulates the expression of several pro-angiogenic factors, like the vascular endothelial growth factor (VEGF-A) [16-18]. Recently, we discovered a further HIF-1-regulated gene, the G-protein estrogen receptor (*GPER*), which contributes to the adaptation to a low oxygen environment in breast cancer cells and in cardiomyocytes [19]. In particular, we found that the cooperation between GPER and HIF-1α upon exposure to hypoxia leads to the up-regulation of the Connective Tissue Growth Factor (*CTGF*) [19], which is a target gene of both HIF-1α [19,20] and GPER [21,22]. Surprisingly, we also assessed that in CAFs derived from breast cancer malignancies GPER acts as a transcription factor promoting the expression of genes involved in cell proliferation and migration [23]. On the basis of these data, GPER-mediated functions may be included among the mechanisms driving the biological responses to hypoxia within the tumor microenvironment.

Considering that hypoxia regulates HIF-1α-dependent expression of both GPER and VEGF in different model systems, in the present study we aimed to evaluate the potential involvement of GPER in the expression and function of VEGF in CAFs and breast cancer cells. In this regard, we demonstrate that HIF-1α/GPER signaling mediates the up-regulation of VEGF as well as the endothelial tube formation, suggesting that this transduction pathway may be involved in the intricate stimulatory responses to hypoxia within the cancer stroma. Our data may open new perspectives towards innovative therapeutic strategies targeting the breast cancer microenvironment.

## Materials and methods

### Reagents

Cobalt chloride (CoCl_2_) and the ROS scavenger N-acetyl-L-cysteine (NAC) were purchased from Sigma-Aldrich Srl (Milan, Italy). PD98059 (PD) was obtained from Calbiochem (Milan, Italy). Human CTGF was purchased from MBL International Corporation, distributed by Eppendorf, (Milan, Italy). Human VEGF was purchased from Peprotech (Rocky Hill, NJ, USA). All compounds were dissolved in DMSO, except NAC, CTGF and VEGF, which were solubilized in water.

### Ethics statement

All procedures conformed to the Helsinki Declaration for the research on humans. The experimental research has been performed with the ethical approval provided by the Ethics Committee of the Regional Hospital in Cosenza, Italy.

### Cell cultures

The SkBr3 breast cancer cells were maintained in RPMI-1640 (Invitrogen, Milan, Italy) without phenol red, supplemented with 10% fetal bovine serum (FBS) and 100 μg/ml penicillin/streptomycin. The murine cardiomyocyte-like cell line HL-1 was kindly provided by Dr. William C. Claycomb (Louisiana State University Medical Center, New Orleans, LA, USA). In particular, the HL-1 cell line was established from a mouse atrial cardiomyocyte tumor excised from an adult female Jackson Laboratory-inbred C57BLy6J mouse (Jackson Laboratory, Bar Harbor, Maine, USA) [24]. HL-1 cells were cultured according to the published protocol [24] in Claycomb medium (JRH Biosciences, Sigma-Aldrich Srl, Milan, Italy) supplemented with 10% FBS (JRH Bioscience, Sigma-Aldrich Srl), 100 μg/ml penicillin/streptomycin (Invitrogen, Milan, Italy), 0.1 mM norepinephrine (Sigma-Aldrich Srl) and 2 mM L-glutamine (Invitrogen). Human umbilical vein endothelial cells (HUVECs) were seeded on collagen-coated flasks (Sigma-Aldrich Srl) and cultured in endothelial growth medium (EGM) (Lonza, Milan, Italy), supplemented with 5% FBS (Lonza, Milan, Italy). All cell lines were grown in a 37°C incubator with 5% CO_2_. For hypoxic stimulation, cells were treated with CoCl_2 _or cultured in the presence of low oxygen tension (2% O_2_) in a HeraCell incubator (ThermoScientific-Heraeus, Milan, Italy). Cells were switched to medium without serum the day before experiments.

### Isolation, cultivation and characterization of CAFs

CAFs were extracted from six invasive mammary ductal carcinomas obtained from mastectomies as previously described [23]. Signed informed consent from all the patients and IRB approval were obtained. In particular, tissues obtained were cut into smaller pieces (1 to 2 mm diameter), placed in digestion solution (400 IU collagenase, 100 IU hyaluronidase and 10% FBS, containing antibiotics and antimycotics solution) and incubated overnight at 37°C. Cells were then separated by differential centrifugation at 90 × g for two minutes. The supernatant containing fibroblasts were centrifuged at 485 × g for eight minutes, the pellet obtained was suspended in fibroblasts growth medium (Medium 199 and Ham's F12 mixed 1:1 and supplemented with 10% FBS and 1% penicillin) and cultured at 37°C, 5% CO_2_. In each patient, a second population of fibroblasts was isolated from a noncancerous breast tissue at least 2 cm from the outer tumor margin. CAFs and fibroblasts were then expanded into two 15-cm Petri dishes and stored as cells passaged for two to three population doublings within a total 7 to 10 days after tissue dissociation. We used CAFs and fibroblasts passaged for up to five population doublings for subsequent experiments to minimize clonal selection and culture stress, which could occur during extended tissue culture. Primary cells cultures of breast fibroblasts were characterized by immunofluorescence. Briefly, cells were incubated with human anti-vimentin (V9) and human anti-cytokeratin 14 (LL001), all antibodies were from Santa Cruz Biotechnology, DBA (Milan, Italy). In order to assess fibroblast activation, we used anti-fibroblast activated protein α (FAPα) antibody (H-56) purchased from Santa Cruz Biotechnology, DBA (Milan, Italy) (data not shown). All experiments were performed in each CAF population obtained from six patients. Data presented were obtained in CAFs derived from one patient; however, results similar to those shown were found in CAFs derived from the other five patients.

### Gene reporter assays

The 2.6 kb *VEGF *promoter-luciferase construct containing full-length *VEGF *promoter sequence (22,361 to +298 bp relative to the transcription start site) used in luciferase assays was a kind gift from Dr. Pal Soumitro (Harvard Medical School, Boston, MA, USA). CAFs or SkBr3 cells (1 × 10^5^) were plated into 24-well dishes with 500 μL/well culture medium containing 10% FBS and transfected for 24 h with control shRNA, shHIF-1α or shGPER. Next, a mixture containing 0.5 μg of reporter plasmid and 10 ng of pRL-TK was transfected within cells. Transfections were performed using FuGENE 6 reagent as recommended by the manufacturer (Roche Diagnostics, Milan, Italy). After 8 h, cells were treated with 100 μM CoCl_2 _or exposed to low oxygen (2% O_2_) for 12 h in serum-free medium. Luciferase activity was measured with the Dual Luciferase Kit (Promega, Milan, Italy) normalized to the internal transfection control provided by Renilla luciferase activity. The normalized relative light unit values obtained from cells treated with vehicle were set as one-fold induction, from which the activity induced by treatments was calculated.

### Gene expression studies

Total RNA was extracted from cell cultures using the Trizol commercial kit (Invitrogen) according to the manufacturer's protocol. RNA was quantified spectrophotometrically and quality was checked by electrophoresis through agarose gels stained with ethidium bromide. Only samples that were not degraded and showed clear 18S and 28S bands under ultraviolet light were used for RT-PCR. Total cDNA was synthesized from the RNA by reverse transcription using the murine leukemia virus reverse transcriptase (Invitrogen) following the protocol provided by the manufacturer. The expression of selected genes was quantified by both real-time RT-PCR using Step One^® ^sequence detection system (Applied Biosystems, Inc., Milan, Italy) and semiquantitative RT-PCR [25]. Gene-specific primers were designed using Primer Express version 2.0 software (Applied Biosystems, Inc.) and are as follows: *HIF-1α *Fwd: 5'-TGCATCTCCATCTTCTACCCAAGT-3' and Rev: 5'-CCGACTGTGAGTGCCACTGT-3'; *GPER *Fwd: 5'-ACACACCTGGGTGGACACAA-3' and Rev: 5'-GGAGCCAGAAGCCACATCTG-3'; *CTGF *Fwd: 5'-ACCTGTGGGATGGGCATCT-3' and Rev: 5'-CAGGCGGCTCTGCTTCTCTA-3'; *VEGF *(human): Fwd: 5'- TGCAGATTATGCGGATCAAACC-3' and Rev: 5'- TGCATTCACATTTGTTGTGCTGTAG-3'; *VEGF *(mouse) Fwd: 5'- GGAGATCCTTCGAGGAGCACTT-3' and Rev: 5'- GGCGATTTA GCAGCAGATATAAGAA-3'; 18S (human, mouse) Fwd: 5'-GGCGTCCCCCAACTTCTTA-3' and Rev: 5'- GGGCATCACAGACCTGTTATT -3. The ribosomal protein *18S *was used as a control gene to obtain normalized values.

For semiquantitative PCR, primers were as follows: *HIF-1α *Fwd: 5'- GCTGATTTGTGAACCCAT TC-3' and Rev: 5'- CTGTACTGTCCTGTGGTGAC-3'; *GPER *Fwd: 5'- CTGGGGAGTTTCCTG TCTGA -3' and Rev: 5'-GCTTGGGAAGTCACATCCAT-3'; *CTGF *Fwd: 5'-ATGGCATGAAGCCAGAGAGT-3' and Rev: 3'-GGTCAGTGAGCACGCTAAAA-3'; *VEGF *(human) Fwd: 5'-GAGCTTCAGGACATTGCTGT-3' and Rev: 5'-AGGAAGGTCAACCACTCACA-3'; VEGF (mouse) Fwd: 5'-CGTGTAAATGTTCCTGCAAA-3' and Rev: 5'-CGTGTAAATGTTCCTGCAAA-3'; *36B4 *(human) Fwd: 5'-CTCAACATCTCCCCCTTCTC-3' and Rev: 5'-CAAATCCCATATCCTCGTCC-3'; *GAPDH *(mouse) Fwd: 5'-ACCACAGTCCATGCCATCAC-3' and Rev: 5'-TCCACCACCCTGTTGCTGTA-3'. *36B4 *and *GAPDH *were used as control genes to obtain normalized values.

### Western blot analysis

To prepare lysates, CAFs were washed in phosphate-buffered saline (PBS) and solubilized with 50 mM Hepes solution, pH 7.4, containing 1% (v/v) Triton X-100, 4 mM EDTA, 1 mM sodium fluoride, 0.1 mM sodium orthovanadate, 2 mM phenylmethanesulfonyl fluoride (PMSF), 10 μg/ml leupeptin and 10 μg/ml aprotinin. Protein concentrations in the supernatant were determined according to the Bradford method. Cell lysates (10 to 50 μg of protein) were electrophoresed through a reducing SDS/10% (w/v) polyacrylamide gel and electroblotted onto a nitrocellulose membrane. Membranes were blocked and incubated with primary polyclonal IgG antibody for HIF-1α (R&D Systems, Inc., Celbio, Milan, Italy), GPER (N-15), CTGF (L-20), phosphorylated ERK1/2 (E-4), ERK2 (C-14), β-actin (C2), b-tubulin (H-235-2) and appropriate secondary HRP-conjugated antibodies, all purchased from Santa Cruz Biotechnology (DBA). The levels of proteins and phosphoproteins were detected with horseradish peroxidase-linked secondary antibodies and revealed using the ECL^® ^System (GE Healthcare, Milan, Italy).

### Gene silencing experiments

Cells were plated onto 10 cm dishes and transfected for 24 h before treatments with a control vector or an independent shRNA sequence for each target gene using Fugene6 (Roche Diagnostics). The shRNA plasmid for HIF-1α and the respective control plasmids were purchased from SABioscience Corporation (Frederick, MD, USA). The silencing of GPER expression was obtained by the construct which we have previously described and used [26].

### Immunofluorescence microscopy

Fifty percent confluent cultured CAFs, SkBr3 and HL-1 cells grown on coverslips were serum-deprived for 24 h and treated for 12 h with 100 μM CoCl_2 _or exposed for 12 h to low oxygen tension (2% O_2_). Then cells were fixed in 4% paraformaldehyde, permeabilized with 0.2% Triton X-100, washed three times with PBS and incubated overnight with a mouse primary antibody against VEGF (C-1) (Santa Cruz Biotechnology, DBA). After incubation, the slides were extensively washed with PBS and incubated with 4′,6-Diamidino-2-phenylindole dihydrochloride (DAPI), (1:1,000), (Sigma-Aldrich Srl) and donkey anti-mouse IgG-FITC (1:300; purchased from Alexa Fluor, Invitrogen). For knockdown experiments, cells were previously transfected for 24 h with shHIF-1α or shGPER and respective negative control plasmids (as described above) and then treated for 12 h with 100 μM CoCl_2_, or cultured under hypoxia as indicated. Leica AF6000 Advanced Fluorescence Imaging System supported by quantification and image processing software Leica Application Suite Advanced Fluorescence (Leica Microsystems CMS, GbH Mannheim, Germany) were used for experiment evaluation.

### Chromatin Immunoprecipitation (ChIP) assay

CAFs were grown in 10-cm dishes to 60 to 70% confluence, serum deprived for 24 h and then treated with vehicle or 100 μM CoCl_2 _for 1 h. Thereafter, cells were cross-linked with 1% formaldehyde and sonicated. Supernatants were immuno-cleared with salmon DNA/protein A-agarose (Upstate Biotechnology, Inc., Lake Placid, NY, USA) and immunoprecipitated with anti-HIF1α or anti-GPER antibody or nonspecific IgG. Pellets were washed, eluted with a buffer consisting of 1% SDS and 0.1 mol/L NaHCO_3_, and digested with proteinase K. DNA was obtained by phenol/chloroform extractions and precipitated with ethanol. The yield of target region DNA in each sample after ChIP was analyzed by real-time PCR. The primer for the -1,216 to -883 region of the human VEGF promoter containing the HRE site is 5'-CACAGACCTTCACAGCCATC-3' (forward hHRE) and 5'-CCCAGCGTAGACAGTTGAGT-3' (reverse hHRE). Data were normalized to the input for the immunoprecipitation. For knockdown experiments CAFs were previously transfected in serum-free medium for 24 h with shHIF-1α or shGPER and the respective control shRNA (as described above) and then treated for 1 h with 100 μM CoCl_2_.

### Migration assay

Migration assays were performed with CAFs in triplicate using Boyden chambers (Costar Transwell, 8 mm polycarbonate membrane, Sigma Aldrich Srl). CAFs were transfected with shRNA constructs directed against HIF-1α, GPER or with an unrelated control shRNA construct in regular growth medium. After 24 h, cells were exposed to low oxygen tension (2% O_2_) or normoxia (20% O_2_) for 6 h in medium without serum. Then, cells were maintained for 24 h in normoxic conditions (20% O_2_) in medium without serum. This experimental condition has been designed in order to reproduce the unbalanced oxygenation occurring within solid tumors, where the irregular blood flow is responsible for hypoxia and reoxygenation phases [27]. CAFs were, therefore, trypsinized and seeded in the upper chambers. CTGF (100 ng/mL) was added to the medium without serum in the bottom wells where applicable. Six hours after seeding, cells on the bottom side of the membrane were fixed and counted.

### Conditioned medium

CAFs were cultured in regular growth medium to 80% confluence. Then, cells were washed twice with PBS and transfected in serum-free RPMI 1640 (Invitrogen) with shHIF-1α, shGPER or control shRNA using Fugene 6 reagent as recommended by the manufacturer (Roche Diagnostics) for 24 h. Subsequently, cells were incubated under normoxic or hypoxic (2% O_2_) conditions for 12 h. Thereafter, the culture supernatant was collected, centrifuged at 16,000 g for five minutes to remove cell debris and used as conditioned medium in HUVECs.

### Protein precipitation with Trichloroacetic acid (TCA)

Conditioned medium from CAFs (see above) was collected, centrifuged at 16,000 g for five minutes to remove cell debris and 10 μg/mL BSA (final concentration) was added as a control for precipitation efficiency. TCA (100% solution) was added to a final concentration of 20% to conditioned media. After a 20-minute incubation on ice, the samples were centrifuged at 31,000 g for 20 minutes (4°C). Pellets were washed with cold acetone, centrifuged at 31,000 g for 30 minutes (4°C), air dried and resuspended in protein lysis buffer (described above). Protein concentrations were estimated using the Bradford Method and immunoblot analysis was performed as described above, using 100 μg protein/lane.

### Tube formation assay

The day before the experiment, confluent HUVECs were starved overnight at 37°C in serum free medium (EBM, Lonza, Milan, Italy). Growth factor-reduced Matrigel^® ^(Cultrex, Trevigen, Inc., Helgerman Court, Gaithersburg, USA) was thawed overnight at 4°C on ice, plated on the bottom of pre-chilled 96-well plates and left at 37°C for 1 h for gelification. Starved HUVECs were collected by enzymatic detachment (0.25% trypsin-EDTA solution, Invitrogen), counted and resuspended in conditioned medium from CAFs. Then, 10,000 cells/well were seeded on Matrigel^® ^and incubated at 37°C. Tube formation was observed starting from 2 h after cell seeding and quantified by using the software NIH ImageJ (National Institutes of Health (NIH), Rockville Pike, Bethesda, Maryland, USA).

### Statistical analysis

Statistical analysis was performed using ANOVA followed by Newman-Keuls' testing to determine differences in means. *P *< 0.05 was considered as statistically significant.

## Results

### Hypoxia induces HIF-1α and GPER expression in CAFs

**Figure 1:**
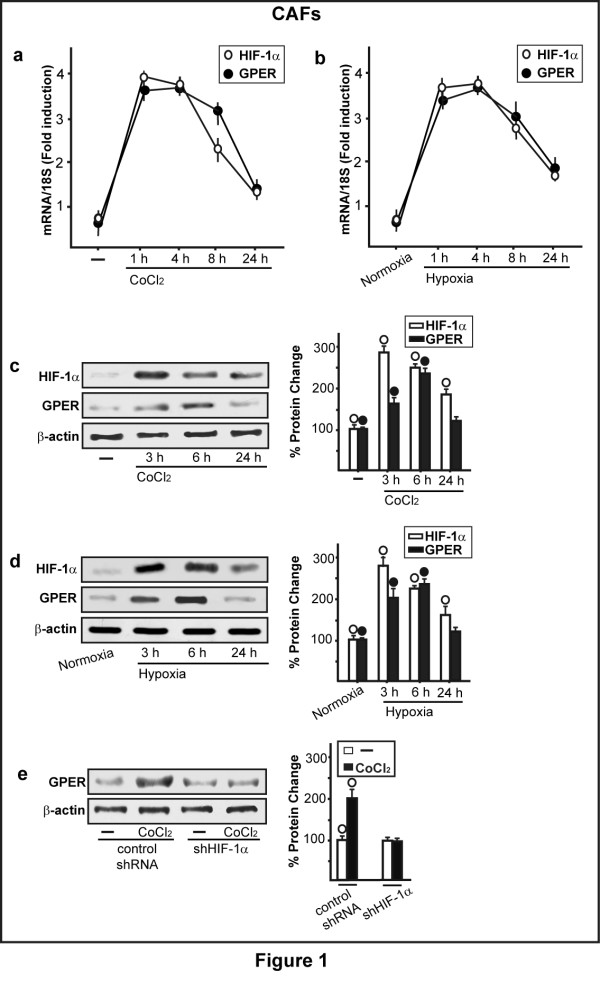
**Hypoxia induces the expression of HIF-1α and GPER in CAFs**. (**a-d**) The exposure to 100 μM CoCl_2 _(a, c) or low oxygen tension (2% O_2 _for 3 h) (b, d) up-regulate the mRNA (a, b) and protein (c, d) expression of *HIF-1α *and *GPER *as evaluated by real-time PCR and immunoblotting, respectively. In RNA experiments, values are normalized to the *18S *expression and shown as fold changes of mRNA expression induced by CoCl_2 _compared to cells treated with vehicle. (**e**) The up-regulation of GPER observed treating CAFs for 6 h with 100 μM CoCl_2 _is abrogated by silencing HIF-1α. Each data point represents the mean ± SD of three independent experiments. Side panel shows densitometric analysis of the blots normalized to β-actin. (○), (●) *P *< 0.05 for cells receiving vehicle (-) or cells cultured under normoxia versus CoCl_2 _treatment or cells cultured under hypoxia.

**Figure 2:**
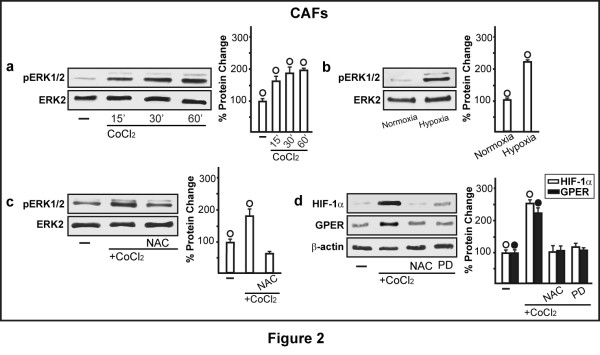
**Hypoxia stimulates the expression of HIF-1α and GPER through ERK1/2 activation in CAFs**. The treatment with 100 μM CoCl_2 _(**a**) or the exposure to low oxygen tension (2% O_2 _for 30 minutes) (**b**) induce ERK1/2 phosphorylation, which is prevented by using 300 μM of the free radical scavenger NAC (**c**). (**d**) Immunoblots of HIF-1α and GPER from CAFs treated for 3 h with vehicle (-) or 100 μM CoCl_2 _alone and in combination with 300 μM NAC or 10 μM ERK1/2 inhibitor PD. Results shown are representative of three independent experiments. Side panels show densitometric analysis of the blots normalized to ERK/2 or β-actin. (○), (●) *P *< 0.05 for cells receiving vehicle (-) or cultured under normoxia vs cells treated with CoCl_2 _or cells cultured under hypoxia.

In order to provide further insight into the response to hypoxia in main components of the tumor microenvironment like CAFs, we began our study showing that hypoxia induces the mRNA expression of both *HIF-1α *and its target gene *GPER*, as ascertained by real time PCR (Figure 1) and semi-quantitative PCR (data not shown). In particular, CAFs were treated from 1 h to 24 h with the hypoxia mimetic agent CoCl_2 _(100 μM) (Figure 1a) or cultured in the presence of low oxygen tension (2% O_2_) (Figure 1b). The induction of *HIF-1α *and *GPER *mRNA expression was paralleled by increased protein levels of these factors in CAFs treated from 1 h to 24 h with 100 μM CoCl_2 _(Figure 1c) or exposed to a low oxygen tension (2% O_2_) (Figure 1d). Next, silencing HIF-1α expression, the induction of GPER by CoCl_2 _was abrogated (Figure 1e, Additional file 1a), indicating that HIF-1α is required for the transcription of GPER in CAFs exposed to hypoxic conditions. In order to assess the transduction signaling involved in the aforementioned responses, we determined the rapid ERK1/2 phosphorylation (from 15 minutes up to 60 minutes) following the treatment with 100 μM CoCl_2 _(Figure 2a). Accordingly, we observed similar effects on ERK1/2 activation culturing CAFs in the presence of low oxygen tension (2% O_2 _for 30 minutes) (Figure 2b). Next, the treatment with 100 μM CoCl_2 _for 30 minutes in the presence of 300 μM of the ROS scavenger NAC did not induce the ERK1/2 phosphorylation (Figure 2c). Moreover, the increase of HIF-1α and GPER observed treating CAFs for 3 h with 100 μM CoCl_2 _was no longer evident in the presence of 300 μM NAC and 10 μM of the MEK inhibitor PD (Figure 2d). On the basis of these results, it could be argued that hypoxia-induced ROS may trigger the ERK1/2 phosphorylation, which is involved in the up-regulation of both HIF-1α and GPER expression.

### HIF-1α and GPER are involved in the expression of VEGF induced by hypoxia

**Figure 3:**
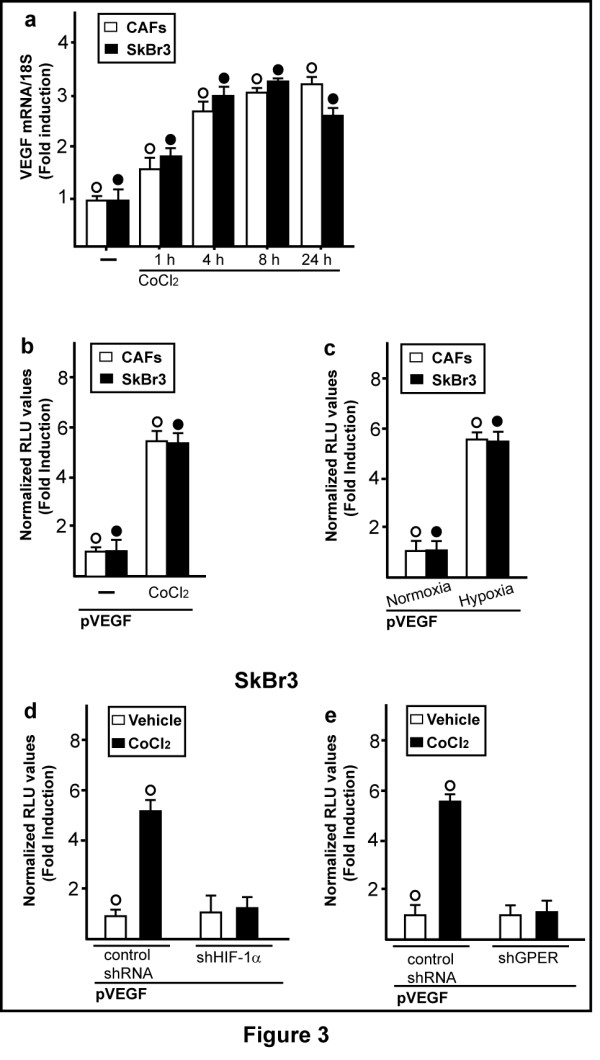
**HIF-1α and GPER are involved in the hypoxia-induced transcriptional activation of VEGF**. (**a**) The mRNA expression of VEGF is up-regulated in CAFs and SkBr3 cells treated with 100 μM CoCl_2_, as evaluated by real-time PCR. Values are normalized to the 18S expression and shown as fold changes of mRNA expression induced by CoCl_2 _compared to cells treated with vehicle (-). Columns, mean of three independent experiments; bars, SD. (**b**) The VEGF promoter plasmid (pVEGF) is transactivated in CAFs and SkBr3 cells treated with 100 μM CoCl_2 _for 12 h (b) or exposed to low oxygen tension (2% O_2_) for 12 h (**c**). The transactivation of the VEGF promoter observed in SkBr3 cells treated for 12 h with 100 μM CoCl_2 _is abrogated by silencing HIF-1α (**d**) or GPER expression (**e**). The luciferase activities were normalized to the internal transfection control and values of cells receiving vehicle or cultured under normoxia were set as one-fold induction upon which the activities induced by CoCl_2 _treatment or hypoxia were calculated. Each data point represents the mean ± SD of three independent experiments performed in triplicate. (○), (●) *P *< 0.05 for cells receiving vehicle (-) or cultured under normoxia vs cells treated with CoCl_2 _or cells cultured under hypoxia.

**Figure 4:**
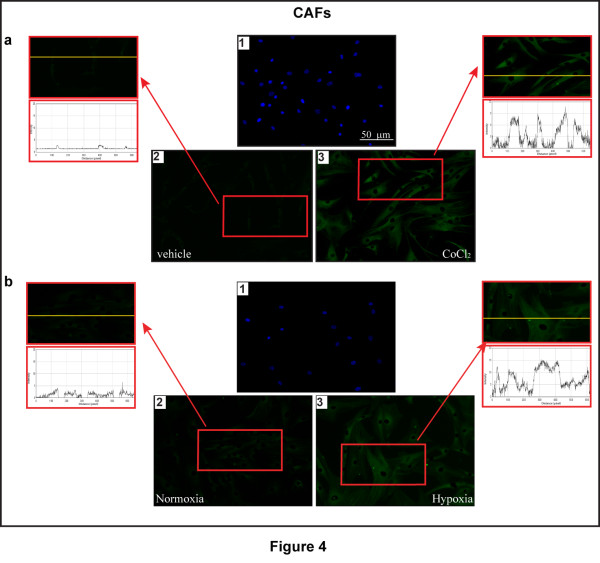
**Hypoxia induces VEGF protein expression in CAFs**. Evaluation of VEGF expression by immunofluorescence assays. CAFs were treated for 12 h with vehicle (**a**2) or 100 μM CoCl_2 _(a3), or cultured under normoxia (**b**2) or low oxygen tension (2% O_2 _for 12 h) (b3), as indicated. VEGF accumulation is evidenced by the green signal. Nuclei were stained by DAPI (blue signal) (a1, b1). For descriptive purposes, each side panel shows the plot profiles obtained at the level of the yellow line of the corresponding inset using the program WCIF Image J for Windows. Note the higher values indicating zones of intense labeling. Images shown are representative of 20 random fields of three independent experiments.

**Figure 5:**
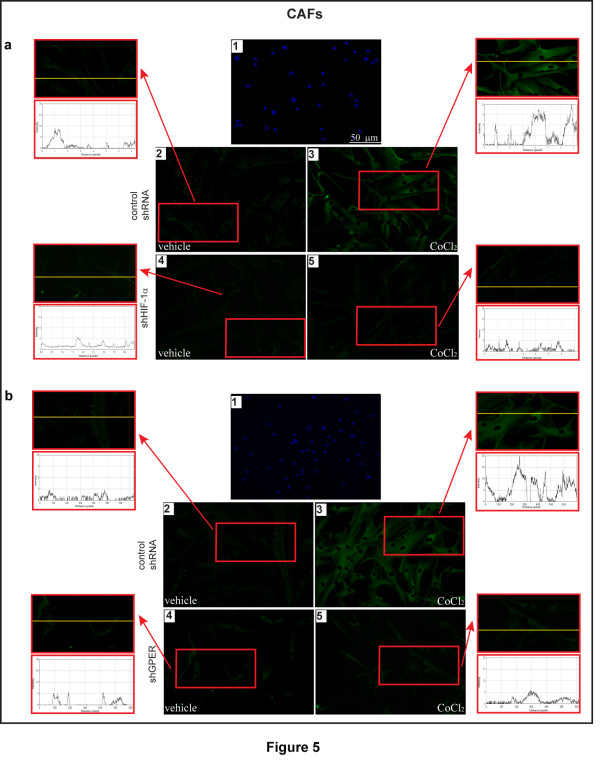
**HIF-1α and GPER mediate the hypoxia-induced expression of VEGF in CAFs**. Evaluation of VEGF expression by immunofluorescent microscopy in CAFs transfected for 24 h with control shRNA (**a**2, a3, **b**2, b3), shHIF-1α (a4, a5) or shGPER (b4, b5). Cells were treated with vehicle or 100 μM CoCl_2 _for 12 h, as indicated and VEGF accumulation is evidenced by the green signal. Nuclei were stained by DAPI (blue signal) (a1, b1). For descriptive purposes, each side panel shows the plot profiles obtained at the level of the yellow line of the corresponding inset using the program WCIF Image J for Windows. Note the higher values indicating zones of intense labeling. Images shown are representative of 20 random fields of three independent experiments.

**Figure 6:**
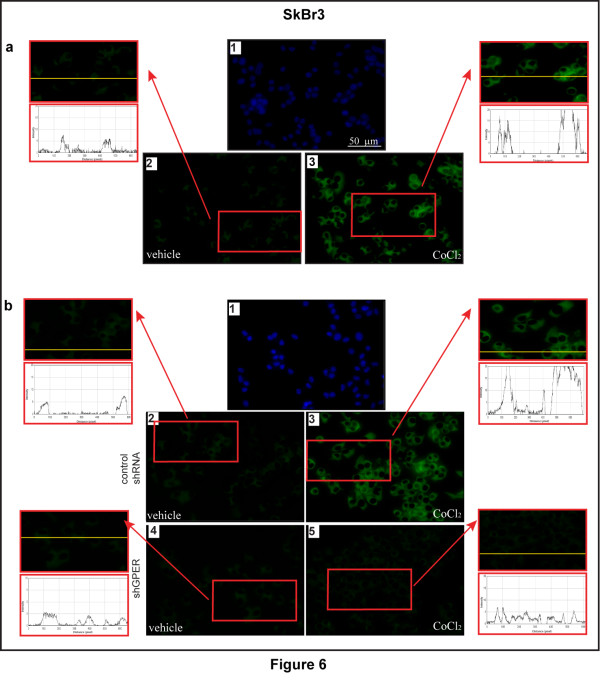
**GPER is involved in VEGF expression by hypoxia in SkBr3 breast cancer cells**. (**a**) SkBr3 cells were treated for 12 h with vehicle (a2) or 100 μM CoCl_2 _(a3), as indicated. VEGF accumulation is evidenced by the green signal. (**b**) SkBr3 cells were transfected for 24 h with control shRNA (b2, b3) or shGPER (b4, b5) and treated with vehicle (b2, b4) or 100 μM CoCl_2 _(b3, b5) for 12 h, as indicated. VEGF accumulation was evidenced by the green signal. Nuclei were stained by DAPI (blue signal) (a1, b1). For descriptive purposes, each side panel shows the plot profiles obtained at the level of the yellow line of the corresponding inset using the program WCIF Image J for Windows. Note the higher values indicating zones of intense labeling. Images shown are representative of 20 random fields of three independent experiments.

**Figure 7:**
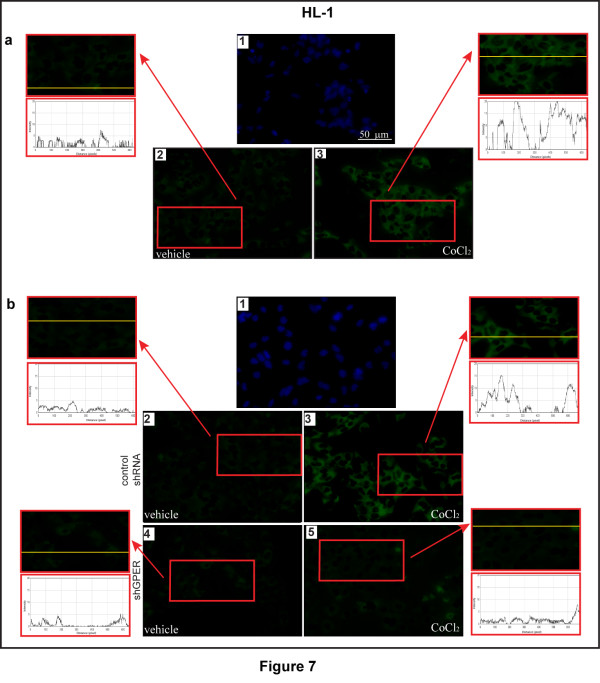
**GPER is involved in VEGF expression by hypoxia in HL-1 murine cardiomyocytes**. (**a**) HL-1 cells were treated for 12 h with vehicle (a2) or 100 μM CoCl_2 _(a3), as indicated. VEGF accumulation is evidenced by the green signal. (**b**) HL-1 cells were transfected for 24 h with control shRNA (b2, b3) or shGPER (b4, b5) and treated with vehicle (b2, b4) or 100 μM CoCl_2 _(b3, b5) for 12 h, as indicated. VEGF accumulation is evidenced by the green signal. Nuclei were stained by DAPI (blue signal) (a1, b1). For descriptive purposes, each side panel shows the plot profiles obtained at the level of the yellow line of the corresponding inset using the program WCIF Image J for Windows. Note the higher values indicating zones of intense labeling. Images shown are representative of 20 random fields of three independent experiments.

Considering the ability of a low oxygen environment to trigger the production of pro-angiogenic factors in solid tumors, we aimed to evaluate whether hypoxia may promote the expression of an important HIF-1α target gene like VEGF. As shown in Figure 3a, the treatment with CoCl_2 _induced the mRNA expression of VEGF in a time-dependent manner in both CAFs and SkBr3 breast cancer cells. Similar results were obtained in a completely different model system, such as HL-1 cardiomyocytes (Additional file 2). Next, CoCl_2 _and a low oxygen tension (2% O_2_) activated a VEGF promoter reporter gene which was transfected in CAFs and SkBr3 cells (Figure 3b, c). Notably, the aforementioned transcriptional response to hypoxia was abrogated knocking-down HIF-1α and GPER expression (Figure 3d, e and Additional file 1c, d). CoCl_2 _and a low oxygen tension (2% O_2_) promoted also VEGF protein expression in CAFs, as evidenced by immunofluorescence experiments (Figure 4). The induction of VEGF protein levels was abolished, silencing HIF-1α and GPER expression (Figure 5 and Additional file 1a, b), further highlighting their involvement in the regulation of VEGF by hypoxia. Analogously, the up-regulation of VEGF protein expression upon exposure to hypoxia in SkBr3 (Figure 6a) and HL-1 cells (Figure 7a) was no longer evident, knocking down the expression of GPER (Figures 6b and 7b and Additional file 1e) and HIF-1 α (data not shown). Taken together, these results suggest that HIF-1α and GPER are involved in the regulation of VEGF expression induced by hypoxia.

### HIF-1α and GPER are both recruited to the VEGF promoter and involved in VEGF-mediated tube formation induced by hypoxia

**Figure 8:**
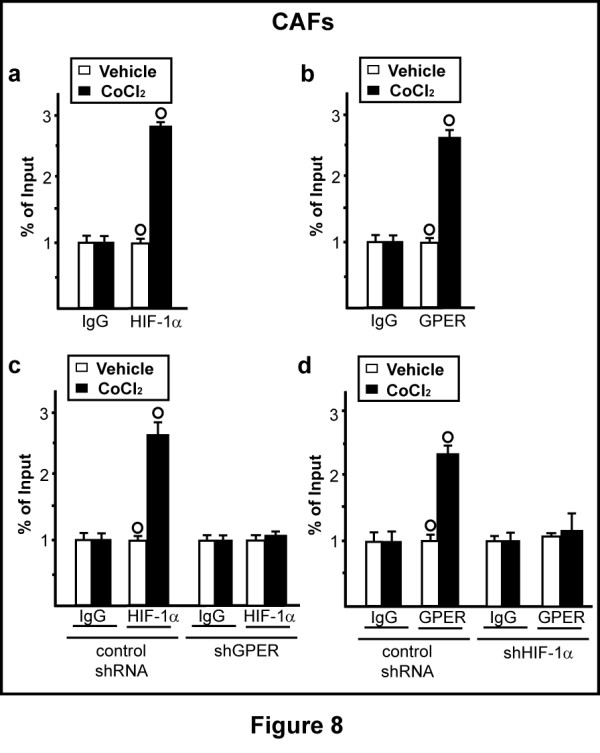
**HIF-1α and GPER are recruited to the VEGF promoter sequence**. CAFs treated for 1 h with 100 μM CoCl_2 _were submitted to the chromatin immunoprecipitation procedure using anti-HIF-1α (**a**) or anti-GPER (**b**) antibodies. For knockdown experiments, CAFs were transfected with control shRNA and shGPER (**c**) or with control shRNA and shHIF-1α (**d**), treated for 1 h with 100 μM CoCl_2 _and then submitted to the chromatin immunoprecipitation procedure using anti-HIF-1α (c) or anti-GPER (d) antibodies. The amplified sequences were evaluated by real-time PCR. Each data point represents the mean ± SD of three independent experiments. (○) *P *< 0.05 for cells receiving vehicle vs CoCl_2 _treatment.

As HIF-1α and GPER are required for the hypoxia-induced VEGF expression, we next determined by ChIP assay that HIF-1α (Figure 8a) and GPER (Figure 8b) are both recruited to the HRE site located within the *VEGF *promoter sequence in CAFs exposed to CoCl_2 _for 1 h. Interestingly, GPER was required for the recruitment of HIF-1α to the promoter of *VEGF*, as evidenced knocking-down the expression of GPER (Figure 8c). In parallel, the silencing of HIF-1α abolished the recruitment of GPER to the HRE site within the *VEGF *promoter sequence (Figure 8d). Altogether, these findings suggest that a functional interplay between HIF-1α and GPER leads to the hypoxia-induced transcription of *VEGF*.

**Figure 9:**
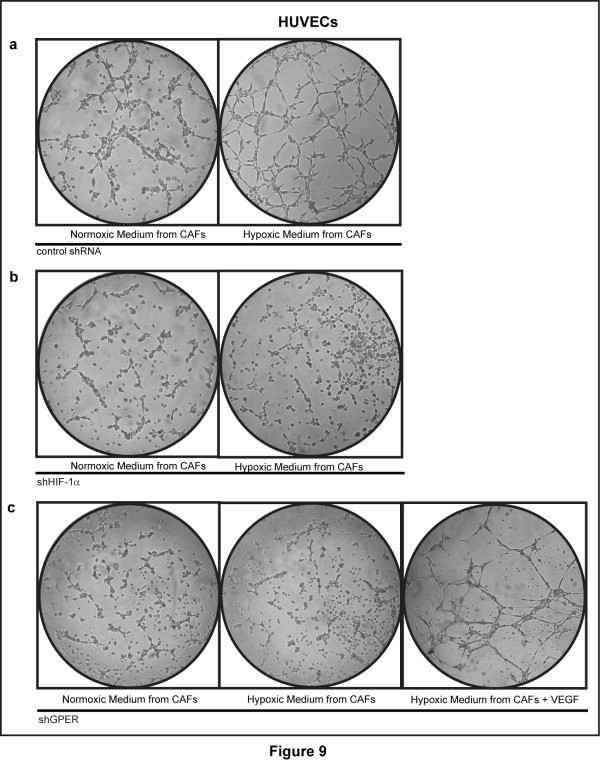
**Involvement of HIF-1α and GPER in hypoxia-induced tube formation**. Tube formation was evaluated in HUVECs cultured for 2 h in medium collected from CAFs which were cultured under normoxia or hypoxia (2% O_2 _for 12 h). To this end, CAFs were transfected with control shRNA (**a**), shHIF-1α (**b**) or shGPER (**c**) and exposed to hypoxia, as indicated. Tube formation is rescued in HUVECs treated with 10 ng/mL VEGF and cultured in medium from CAFs transfected with shGPER and cultured under hypoxia (2% O_2 _for 12 h). Data are representative of three independent experiments performed in triplicate.

On the basis of these data, we then examined whether conditioned medium from hypoxia-stimulated CAFs could promote in HUVECs the formation of tubule-like structures that represent a useful model system for the evaluation of the neoangiogenesis process [28]. In particular, HUVECs were cultured on GFR-Matrigel^®^-coated plates which prevent the formation of capillary-like structures, as previously reported [29]. Notably, HUVECs cultured in normoxic medium from CAFs did not assemble into cord-like structures, while HUVECs grown in medium from CAFs maintained in hypoxic conditions (2% O_2 _for 12 h) displayed a complex ramified network of tubules (Figure 9a). Moreover, in HUVECs cultured with hypoxic medium (2% O_2 _for 12 h) collected from CAFs, which were previously transfected with a shHIF-1α or shGPER, tube formation was no longer observed (Figure 9b, c). The addition of 10 ng/mL VEGF to the hypoxic medium from GPER-silenced CAFs restored the ability to form tubule structures in HUVECs (Figure 9c). The aforementioned findings were quantified and recapitulated in Additional file 3. Next, we determined that the up-regulation of VEGF protein levels in hypoxic medium from CAFs is no longer evident, knocking down HIF-1α and GPER expression (Additional file 4). Hence, these results clearly suggest that VEGF may be considered as a target of the HIF-1α/GPER transduction signaling.

### HIF-1α and GPER are involved in hypoxia-induced expression of CTGF and migration of CAFs

**Figure 10:**
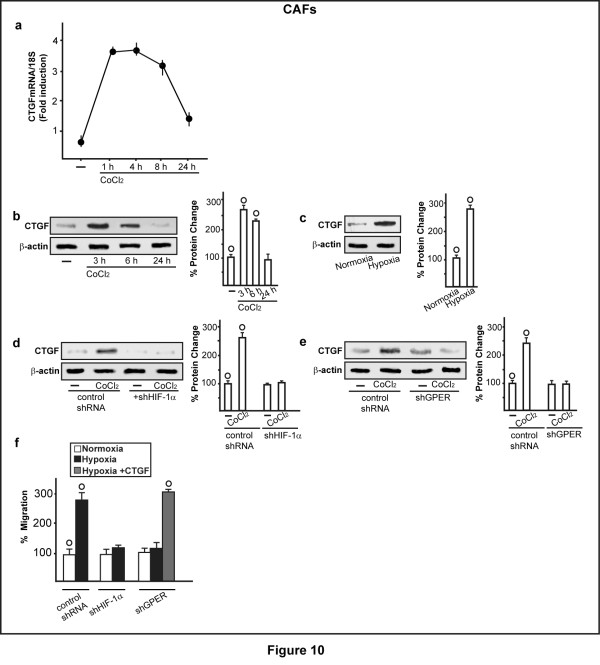
**Involvement of HIF-1α and GPER in hypoxia-induced expression of CTGF and migration of CAFs**. (**a**) The mRNA expression of *CTGF *is induced after the stimulation of CAFs with 100 μM CoCl_2_, as indicated. Values are normalized to the *18S *expression and shown as fold changes of mRNA expression induced by CoCl_2 _compared to cells treated with vehicle. The protein expression of CTGF is induced by 100 μM CoCl_2 _(**b**) or low oxygen tension (2% O_2, _for 4 h) (**c**). The up-regulation of CTGF protein expression observed upon 100 μM CoCl_2 _treatment is abrogated silencing HIF-1α (**d**) or GPER (**e**) expression. The migration of CAFs induced by hypoxia (2% O_2, _for 6 h) is prevented knocking down HIF-1α and GPER expression. Cell migration is rescued in CAFs transfected with shGPER, exposed to hypoxia (2% O_2, _for 6 h) and treated with 100 ng/ml CTGF (**f**). Results shown are representative of three independent experiments. Side panels show densitometric analysis of the blots normalized to β-actin. (○) *P *< 0.05 for cells receiving vehicle (-) or cells cultured under normoxia versus CoCl_2 _treatment or cells cultured under hypoxia.

In order to further corroborate the stimulatory role exerted by HIF-1α/GPER signaling in hypoxic conditions, we turned to a different model system. CoCl_2 _and low oxygen tension (2% O_2_) induced *CTGF *up-regulation at both mRNA (Figure 10a) and protein level (Figure 10b, c) in CAFs. Silencing HIF-1α as well as knocking down GPER expression, the up-regulation of CTGF upon CoCl_2 _was abolished (Figure 10d, e). As a biological counterpart, the migration of CAFs cultured under low oxygen tension (2% O_2_) was prevented, silencing HIF-1α and GPER expression and rescued, adding CTGF (Figure 10f). Collectively, our data may suggest that HIF-1α/GPER signaling mediates different biological outcomes in CAFs exposed to hypoxia.

## Discussion

In the present study, we provide novel evidence regarding the regulation of VEGF, which plays a fundamental role in mediating hypoxia-induced tumor angiogenesis [30,31]. In particular, our results demonstrate that a low oxygen tension stimulates through the ERK1/2 transduction pathway the HIF-1α dependent expression of GPER, which contributes to the regulation of VEGF in both CAFs and breast cancer cells. Moreover, we show that following hypoxic conditions, HIF-1α and GPER are both recruited to the HRE site located within the VEGF promoter region and cooperatively act as a functional complex for the transcription of *VEGF*. As a biological counterpart, we evidence that HIF-1α and GPER mediate endothelial tube formation in HUVECs cultured in medium from CAFs which were previously exposed to hypoxia. Further corroborating these findings, we demonstrate that the cross-talk between HIF-1α and GPER regulates the expression of the migratory factor *CTGF*, which has been acknowledged as a target gene of both HIF-1α and GPER [19,20].

HIF-1 is a master regulator of cellular adaptation to oxygen deprivation and acts as a survival factor in hypoxic tumor environment, mainly activating the transcription of genes involved in glycolytic metabolism, oxygen consumption, cell migration and invasion [15,32]. Besides, hypoxia stimulates through HIF-1 the expression of anti-apoptotic, proliferative and angiogenic factors that drastically change the biological properties of tumor cells towards malignant features [12]. In this context, we have recently demonstrated that *GPER *may be included among the HIF-1 target genes as the up-regulation of GPER induced by hypoxia occurs through the recruitment of HIF-1α to the HRE site located within the GPER promoter sequence in breast cancer cells and cardiomyocytes [19]. In particular, GPER-mediated the antiapoptotic effects exerted by estrogens in hypoxic conditions, suggesting that this receptor may contribute to the adaptation of cancer cells to a low oxygen environment [19].

GPER may be considered as a predictor of cancer malignancy and aggressiveness considering that its expression has been associated with negative clinical features and poor survival rates in diverse types of tumors [33-35]. Therefore, huge efforts are currently underway to unravel the mechanisms that rule its regulation and function. Indeed, the development and characterization of drugs that target key players of cancer progression like GPCRs [36,37], and particularly GPER, are currently innovative topics under investigation [38,39].

In this context, estrogenic GPER signaling has been shown to trigger relevant biological effects like proliferation and migration in diverse cancer cells and in CAFs derived from breast tumors [40-43]. Notably, GPER exhibited the peculiar property to act in CAFs as a transcriptional regulator together with the epidermal growth factor receptor (EGFR), hence leading to the up-regulation of Cyclin D, which has been largely involved in cell proliferation [23]. Increasing evidence has suggested that CAFs may drive cancer phenotype mainly through a paracrine action exerted by the production of various growth factors and chemokines secreted in the tumor microenvironment [3,4,44,45]. Indeed, the stromal contribution to the development of a wide variety of tumors has been supported by animal models of cancer pathogenesis [46,47] and extensive clinical studies [48]. In this vein, it has been shown that malignant cells recruit into the tumor mass diverse stromal components like CAFs, inflammatory and vascular cells that actively cooperate towards cancer progression [46,49-51]. In particular, CAFs elicit in breast carcinomas relevant biological activities, including the stimulation of new blood vessels formation, which closely correlates with cancer growth, metastasis and poor prognosis [52,53]. In this context, our present data add a further mechanism through which the tumor microenvironment may promote angiogenesis, as the HIF-1α/GPER signaling was shown to mediate the up-regulation of VEGF expression in CAFs cultured in hypoxic conditions. Considering that VEGF is one of the most important factors mediating the complex process of angiogenesis [54], its action has been widely involved in the development of many types of tumors, including breast cancer [55]. Accordingly, it has been shown that the ectopic expression of VEGF in MCF-7 breast cancer cells may promote tumor growth *in vivo *[56], whereas the blockade of VEGF has been associated with the growth arrest of breast carcinomas in nude mice [57,58]. Thus, given the crucial role exerted by VEGF in tumor development and prognosis, a great deal of interest is currently addressed to a better understanding of its regulation and function. In this regard, VEGF was shown to be regulated by hypoxia mainly through HIF-1α, which may interact with numerous factors to boost VEGF expression [59,60]. Further extending the current knowledge on the complex regulation of VEGF, our data suggest that GPER may contribute to the HIF-1α mediated transcriptional responses in hypoxic tumor microenvironment towards new blood vessel formation. Notably, an increased VEGF production was recently shown to parallel an elevated GPER expression in endometrial cancer patients with low survival rates [61]. These data in combination with our findings may suggest that in estrogen-dependent cancer cells diverse molecular mechanisms could converge on the production of cytokines and growth factors, promoting an enhancement of malignant epithelial growth and invasion through autocrine and/or paracrine pathways. Interestingly, we have determined that the up-regulation of VEGF relied on HIF-1α/GPER signaling also in HL-1 cardiomyocytes exposed to hypoxia, suggesting that this transduction mechanism may be implicated in additional pathophysiological conditions, such as the hypoxic myocardium. In this regard, it should be mentioned that recent studies have suggested a cardioprotective role exerted by GPER in stressful conditions following hypoxia [62]. These data in combination with the results obtained in the present study may indicate that GPER could elicit cardiotropic effects also through its ability to regulate the expression of VEGF, which mainly contributes to adaptive responses following myocardial ischemic injury [63].

As it concerns the signaling cascades activated by hypoxia, we here demonstrate that the ROS-mediated ERK1/2 activation is involved in the up-regulation of HIF-1α and GPER expression upon low oxygen tension. In this regard, it should be mentioned that ROS play a relevant role in the biological responses to re-oxygenation; however, decreased oxygen levels may be sufficient to trigger HIF-1-dependent gene expression through the ROS production [64-66]. In addition, the rapid responses to hypoxia are mediated by multiple transduction pathways activated also upon GPER stimulation [67,68]. Further studies are needed to better clarify the functional cross-talk between HIF-1α and GPER, particularly following both hypoxic and estrogenic stimulations. For instance, estrogens regulate HIF-1α expression and function in a stressful environment [20], indicating an intricate cooperation between these two main factors mainly involved in tumor progression. Interestingly, in the current investigation, the biological interaction between HIF-1α and GPER upon hypoxic conditions was corroborated by the up-regulation of an important migratory stimulator and GPER target gene like *CTGF*. Accordingly, the migration of CAFs induced by hypoxia was abolished, silencing HIF-1α and GPER expression, hence confirming their contribution to this relevant cell response.

The results obtained in the current study may also disclose a unique ligand-independent action elicited by GPER. This issue could be a further attractive topic which remains to be elucidated in future studies.

Altogether, our findings recapitulate in CAFs exposed to hypoxia the engagement of the HIF-1α/GPER signaling towards the regulation of pivotal genes involved in angiogenic and metastatic processes. Anyway, further studies are required to better define the role exerted by this transduction pathway in diverse pathophysiological conditions as well as the molecular mechanisms driving the biological responses to hypoxia through the functional interaction between HIF-1α and GPER.

## Conclusions

Within the tumor microenvironment exposed to hypoxia, CAFs have been involved in the generation of new blood vessels which mainly support cancer progression. Our current data highlight the stimulatory role exerted by the HIF-1α/GPER signaling in the multistep process of tumor neoangiogenesis fostered by CAFs. Considering the crucial interplay between cancer cells and stroma, the HIF-1α/GPER transduction pathway may be pointed out as a further biological target towards innovative treatments in breast cancer.

## Abbreviations

CAFs: Carcinoma activated fibroblasts; CTGF: connective tissue growth factor; CoCl_2_: cobalt chloride; DAPI: 4′,6-Diamidino-2-phenylindole dihydrochloride; EGFR: epidermal growth factor receptor; EGM: endothelial growth medium; ERK: extracellular signal-regulated kinase; FAPα: fibroblast activated protein α; FBS: fetal bovine serum; GPER: G-Protein estrogen receptor; HIF-1: Hypoxia Inducible Factor-1; HREs: hypoxia-responsive elements; HUVECs: human umbilical vein endothelial cells; N-acetyl-L-cysteine; PBS: phosphate-buffered saline; PMSF: phenylmethanesulfonyl fluoride; TCA: trichloroacetic acid; VEGF: vascular endothelial growth factor.

## Competing interests

The authors declare that they have no competing interests.

## Authors' contributions

EMDF designed and performed the experiments, and wrote the paper. RL, MFS and SM performed the experiments. AC designed the experiments and analyzed data. MM designed the experiments, analyzed data and wrote the paper. All authors read and approved the final manuscript for publication.

## Supplementary Material

Additional file 1 **Evaluation of the HIF-1α and GPER silencing**. Efficacy of HIF-1α (**a**) and GPER (**b**) silencing in CAFs. Efficacy of HIF-1α (**c**) and GPER (**d**) silencing in SkBr3 cells. Efficacy of GPER (e) silencing in HL-1 cells. Side panels show densitometric analysis of the blots normalized to β-actin or β-tubulin, as indicated. Each data point represents the mean ± SD of three independent experiments. Click here for file

Additional file 2 **Hypoxia induces VEGF mRNA expression in HL-1 cells**. The mRNA expression of *VEGF *is induced after the stimulation of CAFs with 100 μM CoCl_2_, as indicated. Values are normalized to the *18S *expression and shown as fold changes of mRNA expression induced by CoCl_2 _compared to cells treated with vehicle. Results shown are representative of three independent experiments. Click here for file

Additional file 3 **Evaluation of tube formation in HUVECs**. Quantification of the number of tubes (**a**), total tube length (**b**) and number of branching points (**c**). Data are representative of three independent experiments performed in triplicate. (○) *P *< 0.05 for HUVECs cultured in normoxic or hypoxic medium from CAFs. Click here for file

Additional file 4 **Evaluation of VEGF expression in conditioned medium from CAFs**. CAFs were transfected with control shRNA, shHIF-1α or shGPER for 24 h and then cultured under normoxia (20% O_2_) or hypoxia (2% O_2_) for 12 h. Culture medium was collected and subjected to protein precipitation using TCA. BSA is shown as the TCA precipitation loading control. Data are representative of three independent experiments performed in triplicate. Side panel shows densitometric analysis of the blots normalized to BSA. (○) *P *< 0.05 for CAFs cultured in normoxia or hypoxia. Click here for file
