# Supplementary figures and images for: HIF-1α/GPER signaling mediates the expression of VEGF induced by hypoxia in breast cancer associated fibroblasts (CAFs)
Source: Breast Cancer Res. 2013 Aug 15;15(4):R64. doi: 10.1186/bcr3458 (PMC3978922; doi:10.1186/bcr3458)

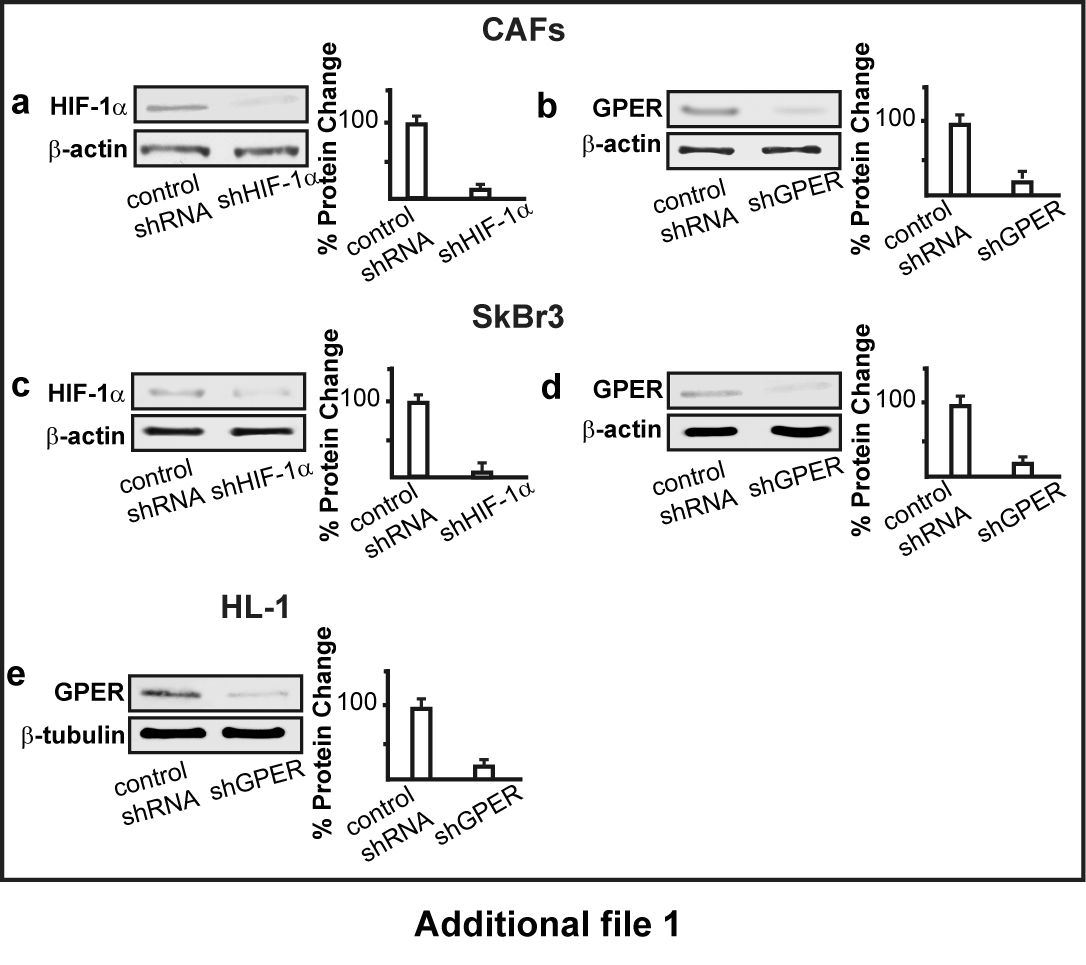

Supplement: Additional file 1 — Evaluation of the HIF-1α and GPER silencing. Efficacy of HIF-1α (a) and GPER (b) silencing in CAFs. Efficacy of HIF-1α (c) and GPER (d) silencing in SkBr3 cells. Efficacy of GPER (e) silencing in HL-1 cells. Side panels show densitometric analysis of the blots normalized to β-actin or β-tubulin, as indicated. Each data point represents the mean ± SD of three independent experiments. [file bcr3458-S1.TIFF]

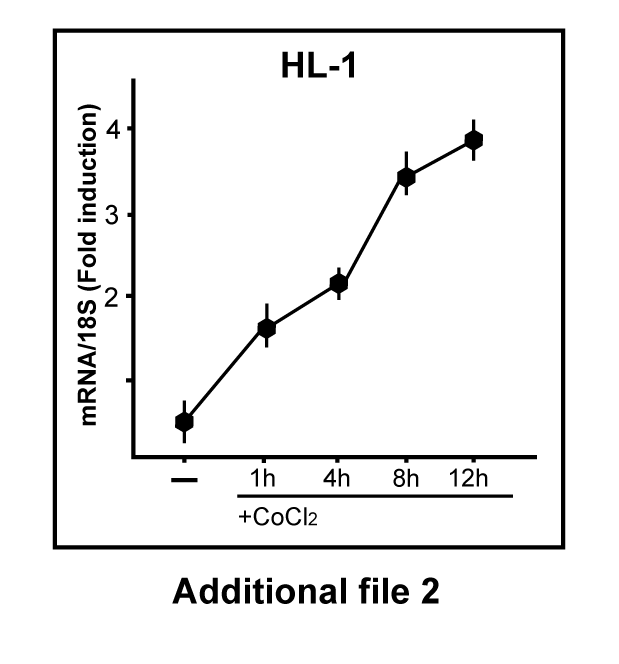

Supplement: Additional file 2 — Hypoxia induces VEGF mRNA expression in HL-1 cells. The mRNA expression of VEGF is induced after the stimulation of CAFs with 100 μM CoCl2, as indicated. Values are normalized to the 18S expression and shown as fold changes of mRNA expression induced by CoCl2 compared to cells treated with vehicle. Results shown are representative of three independent experiments. [file bcr3458-S2.TIFF]

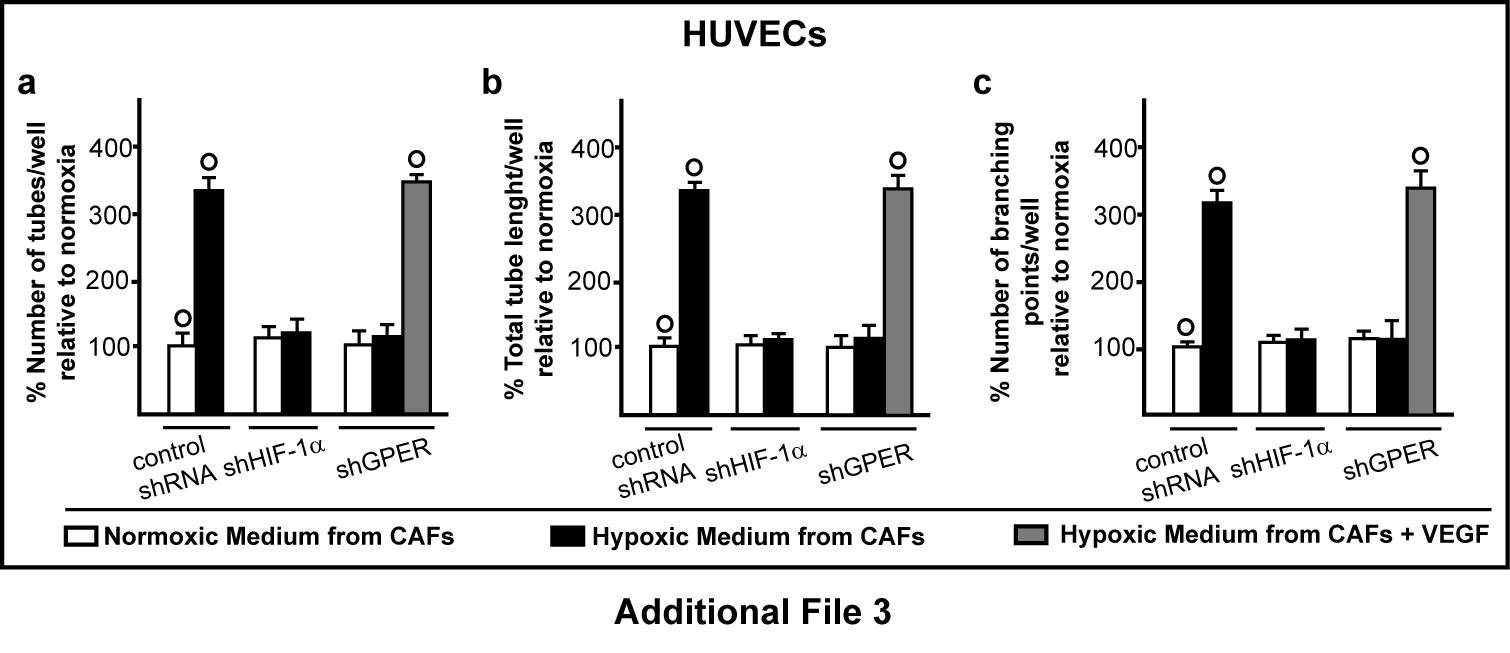

Supplement: Additional file 3 — Evaluation of tube formation in HUVECs. Quantification of the number of tubes (a), total tube length (b) and number of branching points (c). Data are representative of three independent experiments performed in triplicate. (○) P < 0.05 for HUVECs cultured in normoxic or hypoxic medium from CAFs. [file bcr3458-S3.TIFF]

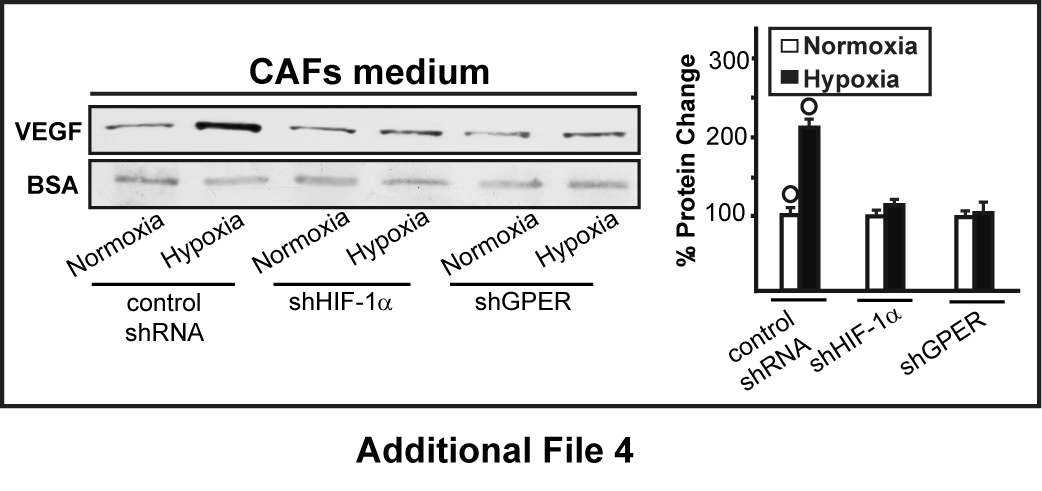

Supplement: Additional file 4 — Evaluation of VEGF expression in conditioned medium from CAFs. CAFs were transfected with control shRNA, shHIF-1α or shGPER for 24 h and then cultured under normoxia (20% O2) or hypoxia (2% O2) for 12 h. Culture medium was collected and subjected to protein precipitation using TCA. BSA is shown as the TCA precipitation loading control. Data are representative of three independent experiments performed in triplicate. Side panel shows densitometric analysis of the blots normalized to BSA. (○) P < 0.05 for CAFs cultured in normoxia or hypoxia. [file bcr3458-S4.TIFF]
